# Novel Phase Shift Microbubbles, MVT-101, Enhance Sonothrombolysis in a Porcine Model of Deep Vein Thrombosis

**DOI:** 10.7150/ntno.120358

**Published:** 2025-10-24

**Authors:** Joel Lusk, Jinwook Kim, Mark Urtz, Gregory Woodhead, Emmanuelle J. Meuillet, Evan C. Unger

**Affiliations:** 1 Microvascular Therapeutics Inc, 1635 E. 18 th Street, Tucson AZ 85719, USA.; 2 University of North Carolina, 109 Mason Farm Road, Chapel Hill, NC 27599, USA.; 3 Synchrony Labs, 3908 Patriot Drive, Suite 170, Durham, NC 27703, USA.; 4 University of Arizona Cancer Center, 1515 N. Campbell Ave, Tucson AZ 85724, USA.

**Keywords:** sonothrombolysis, phase shift microbubbles, porcine model, deep vein thrombosis

## Abstract

**Background:** Estimates suggest that 60,000-100,000 Americans die of Deep Vein Thrombosis / Pulmonary Embolism (DVT/PE) each year. Between 10 to 30% of people with PE will die within one month of diagnosis. Sudden death is the first symptom in about 25% of people who have PE. Among people who have had DVT, one third to one half will have long-term complications (post-thrombotic syndrome) such as swelling, pain, discoloration, and scaling in the affected limb. Microbubble (MB) enhanced sonothrombolysis (SL) has shown promise in treating vascular thrombosis, but MBs are micrometer-sized structures and do not easily permeate clots. We hypothesized that sub-micron structures, e.g., phase shift microbubbles (PSMB or nanodroplets, herein referred as MVT-101) would better permeate clots for more effective SL. We have already shown that MVT-101 (~250 nm in diameter size), easily penetrate the thrombus and demonstrate increased SL efficiency in the rat hindlimb model of microvascular obstruction (MVO).

**Methods:** Bi-lateral occlusive model of DVT was created in the pigs. Endovascular ultrasound was delivered using the EKOS™ Endovascular System and transcutaneous ultrasound (TCUS) was delivered using a diagnostic transducer (GE Vivid E9).

**Results:** We now report that in combination with the EKOS™ Endovascular System, MVT-101 made with perfluoropropane, dissolved occlusive DVT clots in a bi-lateral occlusive porcine model of DVT in combination with (Mean clot resolution = 45.12 ± 46.79 %) or without tissue Plasminogen Activator (tPA) (Mean clot resolution = 36.00 ± 52.27 %) within 60 min of treatment time. We also show that TCUS further SL was observed in the presence of MVT-101 and tPA (Mean clot resolution 76.21 ± 11.86 % with 1000-2000 mg tPA) as compared to tPA alone (Mean clot resolution 16.75 ± 17.00 % with 1000 mg tPA).

**Conclusion:** Both procedures proved to be safe and did not produce PE. In conclusion, MVT-101 holds great potential as therapeutic agent for treating DVT either with TCUS or endovascular ultrasound (EVUS).

## Introduction

Deep Vein Thrombosis (DVT) is a serious disease which affects over 600,000 patients a year in the USA. About 40% of acute DVT develop following major surgery such as cancer or hip/knee replacements. Estimates suggest that 60,000-100,000 Americans die of DVT/Pulmonary Embolism (PE) (venous thromboembolism) each year 1. Between 25% of people with PE will die within one month of diagnosis 1. By far, the major cause of PE is propagation or embolization of clot from DVT into the pulmonary arteries 2. Among people who have had DVT, one third to one half will have long-term complications (post-thrombotic syndrome) such as swelling, pain, discoloration, and scaling in the affected limb 1. While the current standard of care for DVT includes anti-coagulation for mild cases and tissue plasminogen activator (tPA) with endovascular catheter ultrasound for more severe cases 3, most post-operative patients cannot tolerate anti-coagulation or tPA because of the risk of bleeding. There is a clear unmet medical need to develop safer and rapid treatment for acute DVT.

Despite the use of anticoagulant therapy, post-thrombotic syndrome (PTS) develops within 2 years in approximately half of patients with proximal DVT 4-7. PTS commonly causes chronic limb pain and swelling and can progress to cause major disability, leg ulcers, and impaired quality of life 8, 9. Pharmacomechanical catheter-directed thrombolysis (PCDT) delivers a fibrinolytic drug into the thrombus with concomitant thrombus aspiration/agitation 10. The objective of pharmaco-mechanical thrombolysis is to remove thrombus using low-dose fibrinolysis and mechanical agitation, to reduce the risk of PTS while minimizing the risk of bleeding 11-14. The Acute Venous Thrombosis: Thrombus Removal with Adjunctive Catheter-Directed Thrombolysis (ATTRACT) trial demonstrated that the addition of PCDT to anticoagulation did not result in a lower risk of the PTS but did result in a higher risk of major bleeding 15. More recently, ultrasound has also been deployed as an effective technique for PCDT therapy 16, 17. The EKOS™ endovascular ultrasound catheter is FDA approved and uses targeted ultrasonic waves in combination with clot-dissolving drugs in the treatment of pulmonary emboli (PE) 18. Ultrasonic waves delivered from the catheter improve the permeation of tPA into the clot. The thrombolytic dose can be reduced by up to 88-92% with the EKOS catheter as compared to the standard systemic treatment 19, 20.

Further, numerous reports have shown that microbubble contrast agents (MB) enhance the rate of sonolysis with ultrasound 21-24. *In vitro* MB-enhanced sonolysis studies have also shown that clot lysis with this technique does not produce large fragments but rather dissolves clot into constituent components of fibrin, platelets and red blood cells 25. In one *in vitro* study, evaluating MB-enhanced sonolysis with low frequency ultrasound (20 kHz), the residual median microparticle size was 320 nm for ultrasound and MB (range 197-462 nm; p > 0.05) 25. Additionally, in a study examining forward looking transducers for microbubble-mediated sonothrombolysis, a mean thrombolysis rate of 1.4 ± 0.33 mg/min was achieved without the use of a thrombolytic agent 26. In a model of thrombus in the superior vena cava in pigs, MB enhanced sonothrombolysis was successful in dissolving the thrombus and pulmonary scintigraphy showed no evidence of PE 27.

Subsequently, the Microbubbles (MB) and UltraSound accelerated Thrombolysis trial (MUST Trial) for peripheral arterial occlusions was performed to test the efficacy and safety of MB enhanced sonolysis in peripheral arterial occlusions 28, 29. In this trial one vial of Luminity was infused every 15 min while patients were treated for 60 min 28, 29. MB were infused through a McNamara catheter placed intra-arterially in the distal segment of the thrombus 28, 29. The MUST Trial demonstrated that the treatment was feasible and provided evidence of safety without side effects related to the MB, as well as significant improvement in both arterial flow and pain scores 29.

Thrombi have variably porous structures composed of fibrin, platelets and red blood cells 30. Phase Shift Microbubbles (PSMB) are smaller diameter than MB (e.g. 150-350 nm versus 1-3 microns for MB) 23. Our PSMB (herein referred to as MVT-101) are made of octafluoropropane gas encapsulated into a lipid shell made of phospholipids such as dipalmitoylphosphatidylcholine (DPPC), dipalmitoylphosphatidylethanolamine (DPPE), 1,2-dipalmitoyl-sn-glycero-3-phosphoethanolamine-N- [methoxy(polyethylene glycol)-5000] (ammonium salt) (DPPE-MPEG-5000), and 1,2-distearoyl-sn-glycero-3-phosphoethanolamine-N- methoxy(polyethylene glycol)-5000 (DSPE-MPEG-5000) as described in previous studies 31. As the clot progresses from an acute clot with relatively large pores, to the sub-acute or chronic stages where fibrin networks become denser with smaller pores, clot permeability decreases, limiting the penetration of MB 32. Because of their smaller diameter, PSMB better permeate thrombi than MB to afford more effective sonolysis 23, 33-35. An *in vitro* study showed that PSMB enabled sonolysis of retracted, hard clots, which has generally not been achievable with MB sonolysis 36-38. In this study, we tested for the first time both endovascular US (EKOS Endovascular System) and transcutaneous US (GE Vivid E9) in combination with MVT-101 for sonothrombolysis in a porcine model of DVT.

## Material and Methods

### Formulation of MVT-101

For protocols detailing the manufacture and preparation of MVT-101, please refer to previously published methods 31. MVT-101 was first activated and condensed prior to administration. For endovascular treatments 2.6 mL of MVT-101 in 20 mL of saline was administered over 1 hour. For transcutaneous treatments 5.2 mL of MVT-101 in 20 mL of saline was administered over 1 hour. The PSMBs used in this study had an average size of approximately 250-350 nm and as previously described 31.

### DVT porcine model

The animal studies were performed at Synchrony Labs (Durham, North Carolina) under an approved protocol (IACUC 313-01-21). Yorkshire pigs (male and female, ~70kg) were anesthetized with isoflurane gas and ventilated on 100% O_2_ during the procedure. The animals were monitored for blood pressure, heart rate, EKG and pulse oximetry during the procedure. Bilateral iliac vein occlusions were created by inflating a balloon catheter to occlude flow and injecting thrombin, following a protocol adapted from 39. X-ray fluoroscopy was used to introduce balloon catheterization, and venography was performed to visualize the venous anatomy. The clots were allowed to mature for at least 90 minutes and up to 4 hours prior to treatment. Angiography was performed to confirm clot position, size, and degree of occlusion.

### Transcutaneous ultrasound and MVT-101 treatment

For the transcutaneous ultrasound (TCUS) treatment, 4 vials of MVT-101 were infused (administered in a 20 mL volume at a rate of 20 mL/hr) for a total treatment time of 60 minutes. MVT-101 were infused in 6 animals using the multi-side hole Cragg McNamara catheter positioned alongside or within the clot with doses of tPA ranging from 0 to 2,000 micrograms. A GE Vivid E9 system using the 4V-D probe was used for imaging and for sonolysis for the TCUS experiments. Initial imaging was performed with a linear array 9L probe for localization and measurement of flow on Doppler. A Vivid E9 4V-D probe was used for sonolysis. The probe was operated in harmonic mode with a transmit frequency of 1.5 MHz and MI = 1.4 at a frame rate of 46.2-54.1 Hz and depth = 6-12 cm for 60 minutes during infusion of MVT-101 administered intravascularly via syringe infusion pump. The transmit frequency of 1.5 MHz was utilized for PSMB activation. For TCUS treatment, tPA was included within the 20 mL diluted solution of MVT-101. For the control group no treatment was administered.

### Endovascular ultrasound

The EKOS™ catheter was advanced into the clot using 12-cm length acoustic model operating at 2-3 MHz. For the MVT-101, 2 vials were prepared in 20 cc saline and infused through the sheath while ultrasound energy was applied through the catheter. For endovascular ultrasound (EVUS) treatment, tPA was administered through the drug port of the EKOS™ system at a rate of 35 mL/hr with dosages ranging from 0 to 769 micrograms while ultrasound was applied. Various conditions such as tPA, no tPA, and no MVT-101 versus MVT-101 were tested, infusing the same volumes of saline as control.

### Quantification of sonothrombolysis

The length and width of clots were measured using angiography. Post procedure, the percent resolution of the clot was estimated visually by one reviewer. The volume of the clot was also calculated by using the formula for volume of a cylinder, π x length x radius^2^ and the % resolution was obtained using the formula: ((π x length x radius^2^ pre) - (π x length x radius^2^ post treatment))/ (π x length x radius^2^ pretreatment) x 100. The clots were measured onsite post treatment (first blinded reviewer), and a second reviewer (a blinded radiologist) reviewed and measured the clots offsite. The final measurements used for analysis were the data averaged of both onsite and offsite measurements obtained from the two reviewers.

### Whole blood chemistry and hematology analysis

Whole blood chemistry and hematology analysis was performed by Quality Veterinary Laboratory (Davis, CA).

### Statistical analysis

Throughout the manuscript, data were expressed as mean ± standard deviation. For the transcutaneous data, normality and homogeneity of variance were confirmed using the Shapiro-Wilk test and F-tests. One-way ANOVA was then performed to assess differences between groups, followed by Tukey's test to identify individual group differences. For the endovascular data, Shapiro-Wilk and F-tests indicated homogeneity of variance but a non-normal distribution. Therefore, the Kruskal-Wallis test was employed to evaluate significant differences between groups.

## Results

### Sonothrombolysis using transcutaneous ultrasound

**Figure 1:**
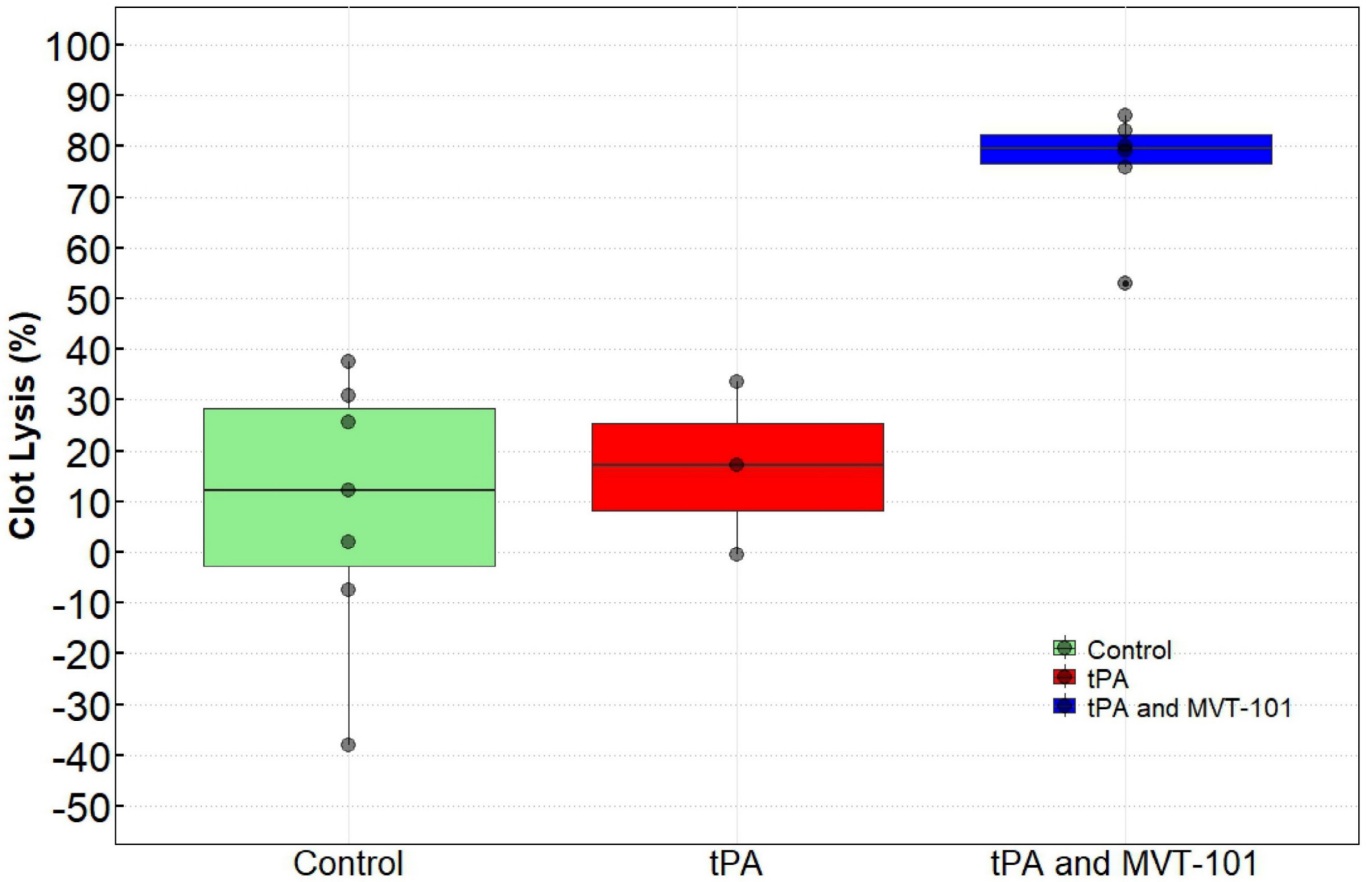
Effects of MVT-101 and tPA in combination with transcutaneous ultrasound.

Pigs that had bilateral occlusive iliac DVT received four doses of MVT-101 that were infused (with 2 doses per 10 mL dose administered at a rate of 20 mL/hr)) for a total treatment time of 60 minutes. There was no significant change in blood pressure, heart rate, or oxygen saturation during the treatment, as shown by the parameters measured and summarized in Table 1. Table 2 details the size the percent reduction in clot size (% resolved) in each pig and Figure 1 represents the averaged results for each treatment group. Of note, TCUS SL effects were observed as early as 20 min post-treatment with MVT-101+tPA (data not shown). While only 8.97±26.17% was calculated with no tPA and no PSMB; the clot resolution increased to 16.75±17.00% in the presence of 1,000 mg tPA and 76.21 ±11.86 with 1,000 mg-2000 mg tPA in combination with MVT-101. The ANOVA test revealed a difference between the groups and a Tukey test revealed that the tPA and MVT-101 group was significantly different from both the tPA condition and the control condition (p=0.0001 as compared to control; p=0.0032 as compared to tPA alone). There was no significant difference between the tPA condition and the control condition (p=0.8467). Blood samples were analyzed for general blood chemistry and comprehensive metabolic panel (Tables 3 and 4) pre- and post-treatment with TCUS, MVT-101 and tPA. Whole blood chemistry and most of the whole blood hematology analyses did not reveal any significant changes between baseline and post-treatment. The sole indicator in the whole blood hematology analysis that changed significantly was the Neutrophil-to-Lymphocytes ratio (NLR), with NLR of ~0.47 pre-treatment and NLR of ~2.97 post-treatment. The increase in this ratio was likely caused by acute DVT 40 since the blood samples were taken right after the treatment (Table 3). We noted also a decrease in albumin (ALB) and total protein (TP) post-treatment as compared to baseline. Fibrinogen was also shown to be unchanged (152±22 ng/ml versus 153±23 ng/ml pre- and post-treatment respectively) indicating no hemorrhagic condition.

We measured several early safety parameters before and after sonothrombolysis treatment. Data are summarized in Table 1. The first set of pre-clinical safety data looked to understand and control the risk of pulmonary emboli via investigation of Pulmonary Artery Pressure (PAP), Pulmonary Capillary Wedge Pressure (PCWP), and Pulmonary Angiogram (PA). Angiograms are shown in Figure 3. All three endpoints can help ensure that pulmonary embolism did not result from the treatment of the DVT with MVT-101. The data collected for PAP, PCWP, and PA did not indicate significant differences between the baseline and end of study results, indicating the absence of pulmonary emboli during treatment with MVT-101. The second set of preclinical safety data looked at pulmonary function via Oxygen Saturation (sO_2_) and heart rate (using Pulse Oximetry). Again, no significant difference was seen between the baseline and end of study results, indicating maintained pulmonary function during treatment with MVT-101. The third set of preclinical safety data looked at the effect of treatment on hemodynamics via investigation of Mean Arterial Pressure (MAP), Systolic Pressure (SYS), and Diastolic Pressures (DIA). All three endpoints can help ensure that adequate blood flow was maintained. The preclinical safety data collected for MAP results did not indicate differences between the baseline and end of study results, indicating adequate blood flow was maintained.

However, SYS and DIA values showed a statistically significant decrease post-treatment with TCUS, MVT-101 and tPA. Ultrasound waves can induce local vasodilation by stimulating endothelial cells to release nitric oxide, a potent vasodilator 41. This relaxation of vascular smooth muscle in the iliac vein and surrounding vessels reduces vascular resistance, lowering both systolic and diastolic pressures. Another explanation may reside in the effective restoration of venous flow. Indeed, successful lysis of a thrombus in the iliac vein reduces obstruction, improving venous return to the heart. While this might initially increase cardiac preload, in some cases, it can lead to a transient drop in systemic vascular resistance as the previously stagnant blood re-enters circulation, potentially diluting vasoconstrictive factors and causing a temporary decrease in blood pressure, as we collected these values within 15 min. post-treatment. Of note, studies in pigs with perfluoropropane MB with same composition used to make MVT-101 (PSMB in this study), have shown that MB cleared by 20 minutes following IV administration. The mean half-life of octafluoropropane in blood after perflutren injection is 1.3 minutes, with the majority eliminated by the lungs within 10 minutes. Sheeran et al showed that perfluoropropane nanodroplets have 3.3x longer circulation time than MB 42. Eventually, perfluoropropane in PSMB will exchange in the circulation and be exhaled by the lungs without metabolism, over a several-fold longer period of time than for perfluoropropane in microbubbles.

### Sonothrombolysis using endovascular ultrasound

**Figure 2:**
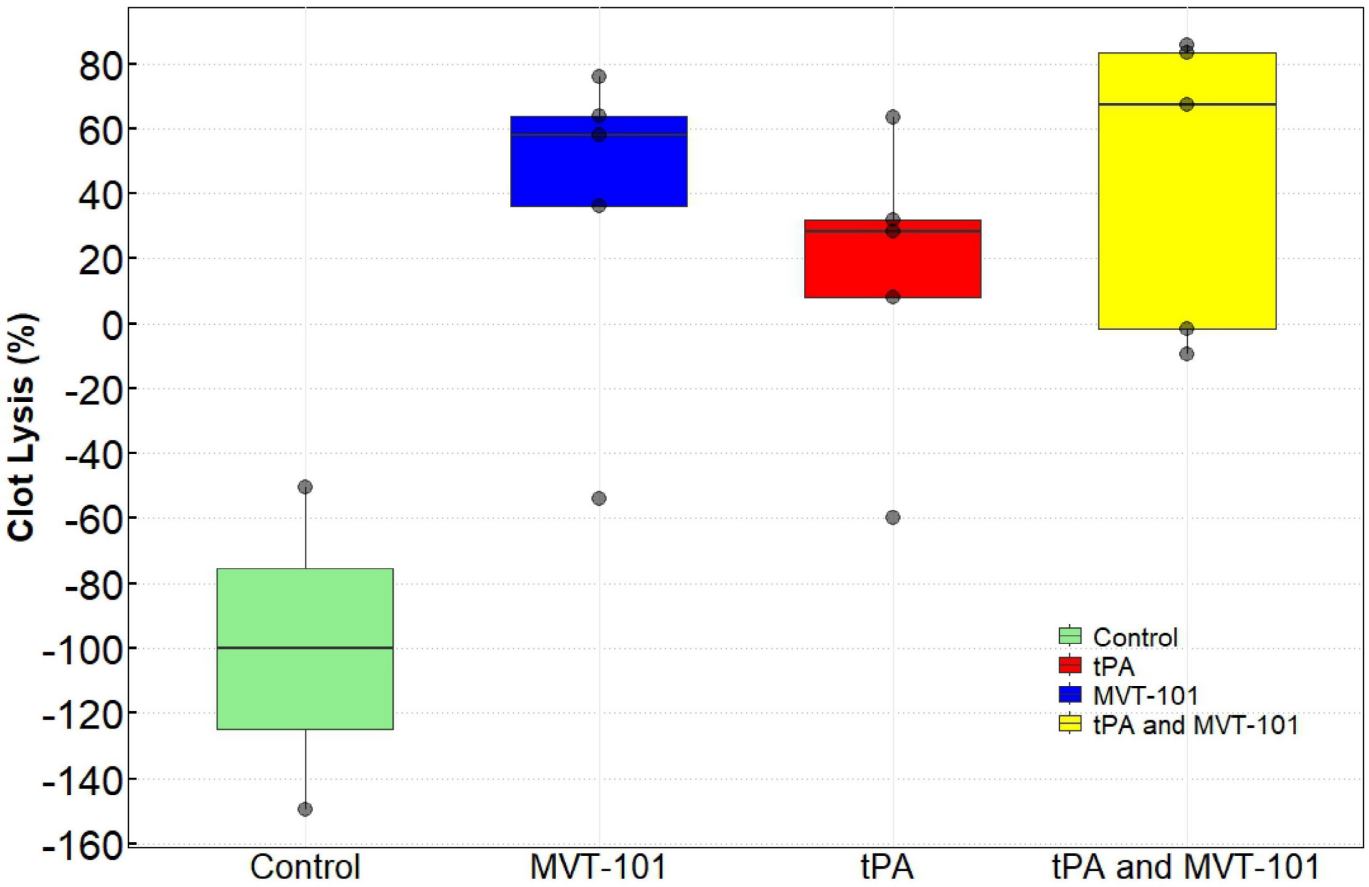
Effects of MVT-101 and Endovascular Ultrasound.

**Figure 3:**
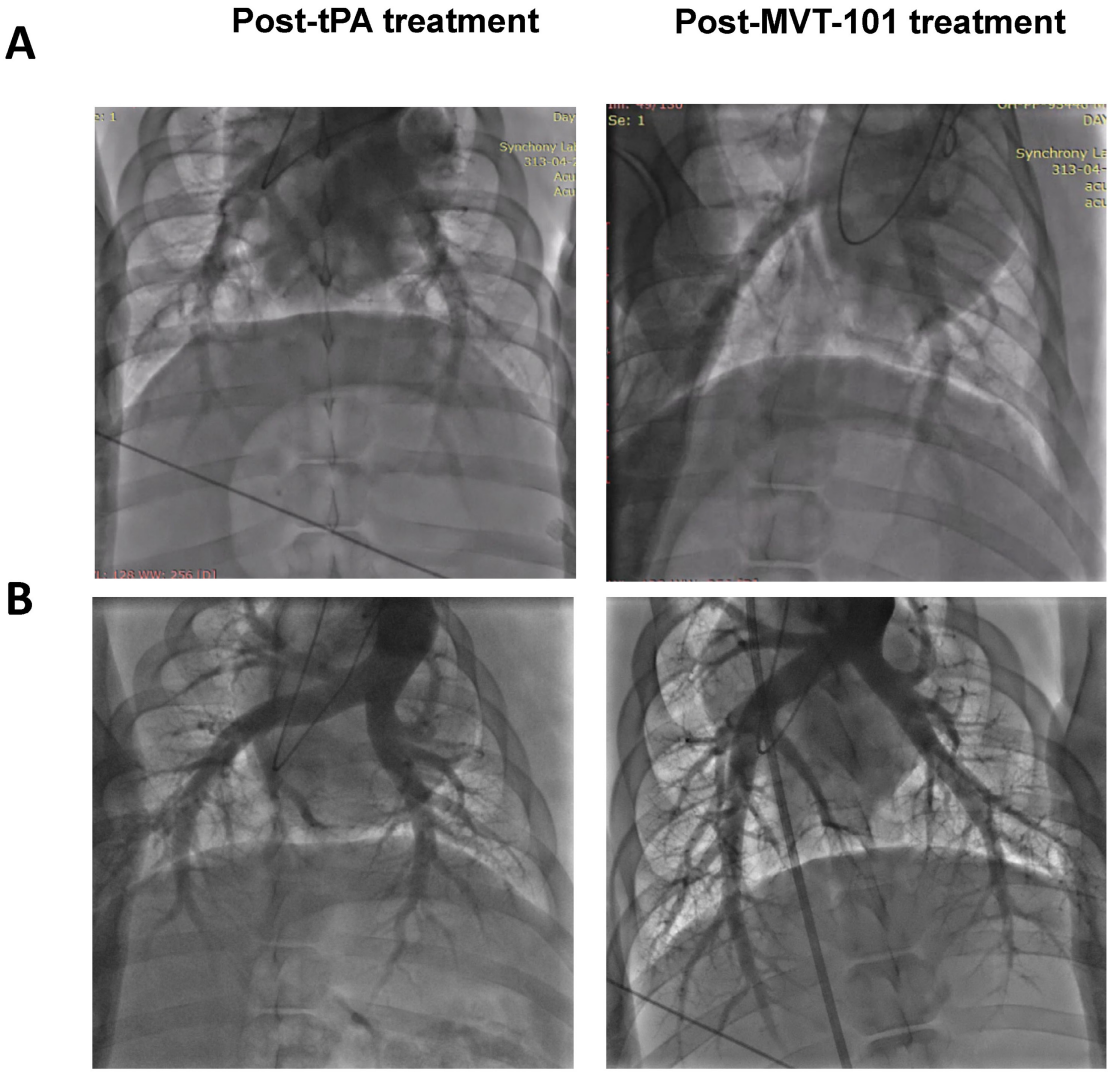
Pulmonary angiograms of post-tPA treatment (left) and post-MVT-101 treatment (right) with Endovascular Ultrasound (Panel A) or Transcutaneous Ultrasound (Panel B).

The data with the EKOS™ endovascular catheter are summarized in Table 5 and data averaged are shown in Figure 2. The mean resolution of the treatment with MVT-101 alone was ~36%, higher than the tPA only condition, which was ~14 %. The highest resolution was obtained with the combination of both MVT-101 and tPA with an average resolution of ~45%. Analysis by the Kruskal-Wallis test showed that there was no significant difference between the different conditions (*p* value of 0.1443). Of note, EVUS + MVT-101 treatment showed efficacy without tPA while the EKOS™ catheter was not effective without tPA. There was a trend to an increase in resolution of the clot when MVT-101 was utilized alone versus tPA alone. We observed a noticeable variability in all treatment conditions using EKOS™ EVUS. Overall, most of the blood compositions summarized in Tables 7 and 8 did not show major changes. As noted for the TCUS treatment, we calculated a NLR of ~0.39 pre- and of ~1.48 post-treatment. We also observed a decrease in albumin (ALB) and total protein (TP) post-treatment as compared to baseline. Parameters and angiograms (Table 6 and Figure 3B) remained unaltered during the course of the treatment with EVUS.

## Conclusion

The comparable efficacy seen with MVT-101 only and with tPA only in combination with the EKOS™ system may indicate that MVT-101 could provide an effective means by which to improve cavitation for the lysis of DVT in patients who would not be able to receive thrombolytic treatment. Transcutaneous ultrasound with MVT-101 and tPA was also consistently efficient at sonothrombolysis and may provide a rapid non-invasive treatment for DVT. We also note that all animals were given IV fluids throughout the procedure to form the clots and during sonothrombolysis. The fluid load and surgical stress (as encountered in the procedures in our experiments) likely causing fluid retention and causing the levels measured in the blood 43. Finally, while preclinical safety and efficacy datasets were collected, we acknowledge that they were obtained from an animal model with acute clot. Acute clots are expected to have high porosity (~50-80%) 44, 45, a loose structure, a better drug penetration. Retracted/hard clots are on the contrary know to have low porosity (~10-30%) 46, 47, be dense and compact, and resistant to both mechanical removal and pharmacologic dissolution. We did not measure the porosity of the clots in our study. In the literature, experiments have confirmed that nanoliposomes with the size around 100 nm are small enough to diffuse freely through the fibrin mesh of thrombi as referenced by Petrokova et al 48. PSMB that are about 1/10 as large as standard MB (average diameter 150-350 nm vs 1-3 microns), and owing to this smaller size, are able to penetrate deeper into microthrombi with nanometer scale fibrin spacing 33, 34, reach tissues beyond the vasculature and deliver medications intracellularly. It is known that the permeability of clots formed *in vivo* can vary by up to five orders of magnitude, with pore sizes that range from 4 to 350 nm according to Wufsus et al 34. In reference to our nanobubbles (PSMB), it has been demonstrated that they can achieve thrombolysis of acute and hard/retracted clots 36-38. To our knowledge, there is not an animal model that exists that has been able to reproducibly model chronic DVT. The current model for chronic DVT in pigs that has been cited has a high attrition rate 49 and would not reliably illustrate the physiology of chronic DVT.

## Supplementary Material

Supplementary figures.

## Figures and Tables

**Table 1:** ** Parameters at baseline and post-treatment with transcutaneous ultrasound, MVT-101 and tPA.** Data are the mean ± standard deviations, number of pigs, n =5, and * for p<0.05; ** for p<0.01 and *** for p<0.005.

Parameters	Baseline	End of Study	*p* value
Pulmonary Artery Pressure (PAP)	20.0±4.3	18.8±6.2	0.72
Pulmonary Capillary Wedge pressure (PCW)	11.2±3.4	9.8±3.0	0.51
Oxygen Saturation (sO_2_)	99.3±1.5	99.3±1.5	0.82
Heart Rate (Pulse Oximetry)	85.3±9.8	91.5±17.5	0.55
Mean arterial pressure (MAP)	70.0±8.5	66.3±7.4	0.53
Systolic pressure (SYS)***	89.0±16.2	53.8±7.6	0.002
Diastolic pressure (DIA)**	74.6±6.9	55.2±9.4	0.006

**Table 2:** ** Transcutaneous ultrasound dataset.** Dataset obtained for each pig. L, for left; R, for right; tPA for tissue plasminogen Activator. NA, for not available.

Pig	Vein	Resolved (%)	Treatment	Clot Mature Time (minutes)	tPA (µg)
**8**	L iliac vein	80.11	tPA with MVT-101	102	2000
**10**	L iliac vein	83.06	tPA with MVT-101	106	2000
**11**	L iliac vein	79.03	tPA with MVT-101	111	2000
**9**	R iliac vein	53.06	tPA with MVT-101	112	2000
**12**	R iliac vein	86.05	tPA with MVT-101	107	1000
**13**	L iliac vein	75.96	tPA with MVT-101	108	1000
**8**	R iliac vein	-37.98	Control	NA	0
**10**	R iliac vein	1.95	Control	NA	0
**13**	R iliac vein	25.73	Control	NA	0
**12**	L iliac vein	-7.50	Control	NA	0
**11**	R iliac vein	37.48	Control	NA	0
**9**	L iliac vein	30.88	Control	NA	0
**14**	R iliac vein	12.26	Control	NA	0
**29**	R iliac vein	17.13	tPA	110	1000
**29**	L iliac vein	33.56	tPA	215	1000
**30**	R iliac vein	-0.44	tPA	133	1000

**Table 3:** ** Whole blood hematology analysis at baseline and post-treatment with transcutaneous ultrasound, MVT-101 and tPA.** Data are the means ± standard deviations, n=5 pigs, * for p<0.05; ** for p<0.01 and *** for p<0.005. NA, for not available.

Test	Units	Baseline	Post-treatment	*p* value
**WBC**	**x10³ /uL**	**17.5±1.8**	**18.4±3.9**	**0.99**
**RBC**	**x10^6^/uL**	**5.5±0.9**	**5.1±0.4**	**0.59**
**HGB**	**g /dL**	**10.2±1.5**	**9.4±0.6**	**0.51**
**HCT**	**%**	**31.4±4.5**	**28.8±2.1**	**0.47**
**MCV**	**fL**	**57.5±1.6**	**57.1±1.7**	**0.78**
**MCH**	**pg**	**18.7±0.6**	**18.7±0.8**	**0.97**
**MCHC**	**g / dL**	**32.6±0.2**	**32.8±0.6**	**0.72**
**RDW**	**%**	**14.8±0.6**	**14.7±0.6**	**0.88**
**PLT**	**x10³ /uL**	**213.8±49.5**	**197.8±54.5**	**0.85**
**MPV**	**fL**	**9.4±0.8**	**8.9±0.9**	**0.37**
**%NEUT**	**%**	**29.8±10.2**	**72.6±8.3**	0.002, **
**%LYMPHS**	**%**	**63.8±9.6**	**24.4±8.0**	0.002, **
**%MONO**	**%**	**2.0±0.9**	**0.4±0.5**	0.01, **
**%EOS**	**%**	**3.8±1.1**	**2.4±1.1**	**0.31**
**%BASO**	**%**	**0.4±0.5**	**0.2±0.4**	**0.35**
**%BANDS**	**%**	**0**	**0**	**NA**
**#NEUT**	**x10³ /uL**	**5.3±2.2**	**13.5±3.8**	0.02, *
**#LYMPHS**	**x10³ /uL**	**11.1±1.8**	**4.3±1.1**	0.002, **
**#MONO**	**x10³ /uL**	**0.3±0.1**	**0.1±0.1**	0.01, *
**#EOS**	**x10³ /uL**	**0.6±0.2**	**0.5±0.3**	**0.47**
**#BASO**	**x10³ /uL**	**0.1±0.1**	**0**	**0.35**
**#BANDS**	**x10³ /uL**	**0**	**0**	**0**

**Table 4:** ** Whole blood chemistry analysis at baseline and post-treatment with transcutaneous ultrasound, MVT-101 and tPA.** Data are the means ± standard deviations, n=5 pigs, * for p<0.05; ** for p<0.01 and *** for p<0.005.

Test	Units	Baseline	Post-treatment	*p* value
ALB	g/dL	2.9±0.1	2.5±0.1	<0.005, ***
TP	g/dL	4.9±0.2	4.3±0.1	<0.005, ***
ALP	U/L	97.5±33.1	83.8±29.2	0.82
ALT	U/L	24.2±6.2	20.0±5.2	0.49
AST	U/L	16.3±2.1	14.8±3.3	0.48
CK	U/L	627±254	640±275	0.85
TBIL	mg/dL	0.1±0.04	0.2±0.1	0.19
DBIL	mg/dL	0.03±0.04	0.1±0.1	0.06
BUN	mg/dL	5.3±0.9	6.6±1.1	0.13
CREAT	mg/dL	1.3±0.1	1.3±0.1	0.29
CA	mg/dL	9.3±0.2	9.0±0.2	0.03, *
CHOL	mg/dL	60.3±7.8	52.6±9.3	0.29
GLU	mg/dL	91.2±33.4	124.2±10.8	0.15
PHOS	mg/dL	7.9±0.6	7.1±0.4	0.07
TCO2	mEq/L	28.3±2.2	26.2±1.6	0.12
NEUT	mEq/L	140.7±1.8	136.6±1.8	0.01, *
K	mEq/L	4.2±0.3	4.1±0.2	0.29
CL	mEq/L	102.8±1.2	100.0±1.2	0.01, *
GLOB	g/dL	2.1±0.1	1.8±0.1	0.01, *
A/G	Ratio	1.4±0.1	1.4±0.1	0.72
B/C	Ratio	4.2±0.7	5.2±0.4	0.05, *
IBIL	mg/dL	0.08±0.07	0.10±0.44	0.61
ANION	mEq/L	13.7±1.9	14.4±1.5	0.31

**Table 5:** ** Endovascular ultrasound dataset.** Dataset obtained for each pig. L, for left; R, for right; tPA for tissue plasminogen Activator.

Pig	Vein	Resolved (%)	Treatment	Clot Mature Time (minutes)	TPA (µg)
15	L iliac vein	83.46	tPA with MVT-101	120	769
17	L iliac vein	85.76	tPA with MVT-101	110	500
20	R iliac vein	-9.39	tPA with MVT-101	178	500
21	L iliac vein	-1.50	tPA with MVT-101	179	500
22	R iliac vein	67.24	tPA with MVT-101	183	500
15	R iliac vein	57.90	MVT-101	99	0
16	R iliac vein	36.25	MVT-101	101	0
21	R iliac vein	75.92	MVT-101	98	0
22	L iliac vein	63.82	MVT-101	102	0
23	L iliac vein	-53.90	MVT-101	191	0
18	L iliac vein	28.43	tPA	112	500
20	L iliac vein	63.57	tPA	99	500
24	R iliac vein	32.07	tPA	111	500
24	L iliac vein	7.99	tPA	111	500
17	R iliac vein	-59.87	tPA	105	500
19	R iliac vein	-149.60	Control	106	0
16	L iliac vein	-50.49	Control	101	0

**Table 6:** ** Parameters at baseline and post-treatment with endovascular ultrasound, MVT-101 and tPA.** Data are the mean ± standard deviations, number of pigs, n =5, and * for p<0.05; ** for p<0.01 and *** for p<0.005.

Parameters	Baseline	End of Study	*p* and n
Pulmonary Artery Pressure (PAP)	21.0±2.2	18.7±1.3	0.25
Pulmonary Capillary Wedge pressure (PCW)	16.3±2.1	15.0±3.3	0.65
Oxygen Saturation (sO_2_)	99.7±0.5	99.7±0.8	1.00
Heart Rate (Pulse Oximetry)	95.2±9.1	99.2±19.4	0.66
Mean arterial pressure (MAP)	67.3±6.7	62.0±7.3	0.22
Systolic pressure (SYS)	90.2±7.5	78.5±10.3	0.14
Diastolic pressure (DIA)	53.2±5.3	49.7±5.5	0.33

**Table 7:** ** Blood hematology analyses pre- and post-endovascular US treatment** (n=4 pigs, * for p<0.05)

Test	Units	Baseline	Post-treatment	p value
ALB	g/dL	3.2±0.1	2.9±0.1	0.02, *
TP	g/dL	5.4±0.1	4.8±0.6	0.01, *
ALP	U/L	126.7±35.5	118.5±34.0	0.76
ALT	U/L	28.7±3.9	25.0±2.9	0.21
AST	U/L	18.3±1.8	17.0±2.2	0.43
CK	U/L	487.5±131.1	558.0±184.4	0.57
TBIL	mg/dL	0.15±0.08	0.20±0.06	1
DBIL	mg/dL	0.05±0.07	0.10±0.06	1
BUN	mg/dL	5.0±1.6	6.0±1.8	0.47
CREAT	mg/dL	1.3±0.2	1.1±0.2	0.42
CA	mg/dL	9.7±0.3	9.4±0.3	0.21
CHOL	mg/dL	71.5±10.4	67.5±6.6	0.58
GLU	mg/dL	125.7±21.6	119.5±26.5	0.74
PHOS	mg/dL	7.6±0.4	7.4±0.3	0.43
TCO2	mEq/L	30.0±1.2	28.0±1.4	0.09
NEUT	mEq/L	139.3±1.9	138.3±2.5	0.57
K	mEq/L	4.8±0.3	4.6±0.7	0.59
CL	mEq/L	102.8±1.3	103.8±3.3	0.60
GLOB	g/dL	2.1±0.2	1.9±0.3	0.16
A/G	Ratio	1.5±0.2	1.7±0.3	0.52
B/C	Ratio	4.3±1.5	5.5±2.1	0.39
IBIL	mg/dL	0.10±0.07	0.30±0.46	0.37
ANION	mEq/L	11.5±1.1	11.5±1.3	1.00

**Table 8:** ** Blood chemistry analysis pre- and post-treatment with endovascular ultrasound, with MVT-101 and tPA.** Data are the mean ± standard deviations, n=4 pigs, * for p<0.05; ** for p<0.01 and *** for p<0.005.

Test	Units	Baseline	Post-treatment	*p* value
WBC	x10³ /uL	18.7±1.9	14.2±0.6	0.008,**
RBC	X10**^6^**/uL	6.2±0.5	5.5±0.2	0.09
HGB	g /dL	11.2±0.5	10.0±0.5	0.02, *
HCT	%	34.4±1.5	30.8±1.6	0.02, *
MCV	fL	56.1±2.7	55.9±3.0	0.91
MCH	pg	18.2±0.8	18.0±1.1	0.77
MCHC	g / dL	32.5±0.3	32.3±0.2	0.27
RDW	%	14.5±0.9	14.6±1.1	0.90
PLT	x10³ /uL	203.5±37.9	186.8±25.2	0.53
MPV	fL	8.8±0.9	8.1±0.6	0.30
%NEUT	%	26.5±2.7	58.5±1.3	<0.0001,***
%LYMPHS	%	67.0±3.5	39.5±1.29	<0.0001, ***
%MONO	%	3.3±0.8	1.0±0.0	0.003, ***
%EOS	%	3.0±0.7	1.3±0.5	0.001, ***
%BASO	%	0.3±0.4	0.0±0.0	0.35
%BANDS	%	0±0	0±0	NA
#NEUT	x10³ /uL	4.9±0.9	8.3±0.3	0.0008, ***
#LYMPHS	x10³ /uL	12.4±1.1	5.6±0.3	<0.0001, ***
#MONO	x10³ /uL	0.6±0.2	0.10±0.01	0.003, ***
#EOS	x10³ /uL	0.6±0.2	0.20±0.08	0.001, ***
#BASO	x10³ /uL	0.05±0.09	0.0±0.0	0.35
#BANDS	x10³ /uL	0±0	0±0	NA

## Abbreviations

ALB: Albumin
ALP: Alkaline phosphatase
AALT: Alanine aminotransferase
AST: Aspartate aminotransferase
A/G: Albumin/Globulin ratio
ANION: Anionic
BANDS: Band Neutrophils
BASO: Basophils
B/C: Bilirubin/Creatinine ratio
BUN: Blood urea nitrogen
CA: Carbohydrate antigen
C: Creatinine
CREAT: Creatinine
CHOL: Cholesterol
CL: Total cholesterol
DIA: Diastolic Pressures
DBIL: Direct bilirubin
DVT: Deep Vein Thrombosis
EOS: Eosinophils
EVUS: Endovascular Ultrasound
GLU: glucose
GLOB: globulin
HCT: Hematocrit
HGB: Hemoglobin
IBIL: Intrahepatal bilirubin
K: Potassium
LYMPHS: Lymphocytes
MAP: Mean Arterial Pressure
MCV: Mean Corpuscular Volume
MCH: Mean Corpuscular Hemoglobin
MCHC: Mean Corpuscular Hemoglobin Concentration
MB: Microbubbles
MPV: Mean Platelet Count
MVO: Microvascular Obstruction
NEUT: Neutrophils
NLR: Neutrophil-to-Lymphocytes ratio
PA: Pulmonary Angiogram
PAP: Pulmonary Artery Pressure
PCWP: Pulmonary Capillary Wedge Pressure
PCDT: Pharmacomechanical catheter-directed thrombolysis
PE: Pulmonary Emboli
PHOS: phosphate
PLT: Platelet Count
PSMB: Phase Shift Microbubbles
PTS: Post-thrombotic Syndrome
RBC: Red Blood Cells
RDW: Red Cell Distribution Width
SYS: Systolic Pressure
sO_2_: Oxygen Saturation
SL: Sonothrombolysis
MONO: Monocytes
TCO2: Total carbon dioxide
TCUS: transcutaneous ultrasound
TBIL: Total bilirubin
TP: Total Protein
tPA: tissue Plasminogen Activator
US: Ultrasound
WBC: Whole Blood Cells
