# Supplementary material for: Novel Phase Shift Microbubbles, MVT-101, Enhance Sonothrombolysis in a Porcine Model of Deep Vein Thrombosis
Source: Nanotheranostics. 2025 Oct 24;9(3):289–98. doi: 10.7150/ntno.120358 (PMC12595264; doi:10.7150/ntno.120358)

Representative Venograms

Endovascular

Pig #	Location	Treatment	Ultrasound
16	Right Iliac Vein	MVT-101	Endovascular

Pre Treatment

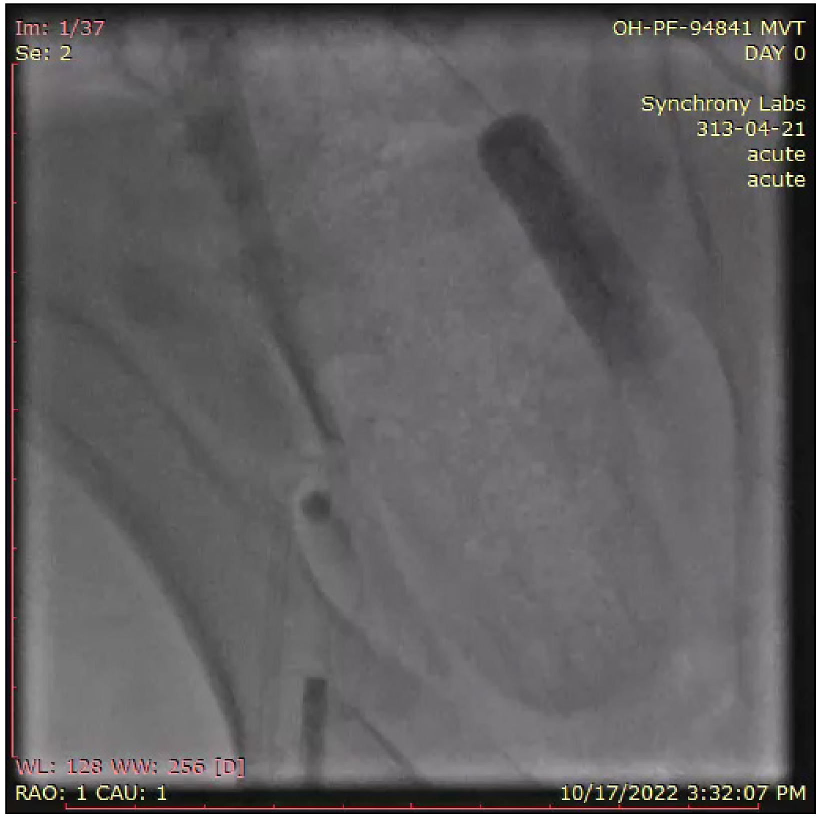

Post Treatment

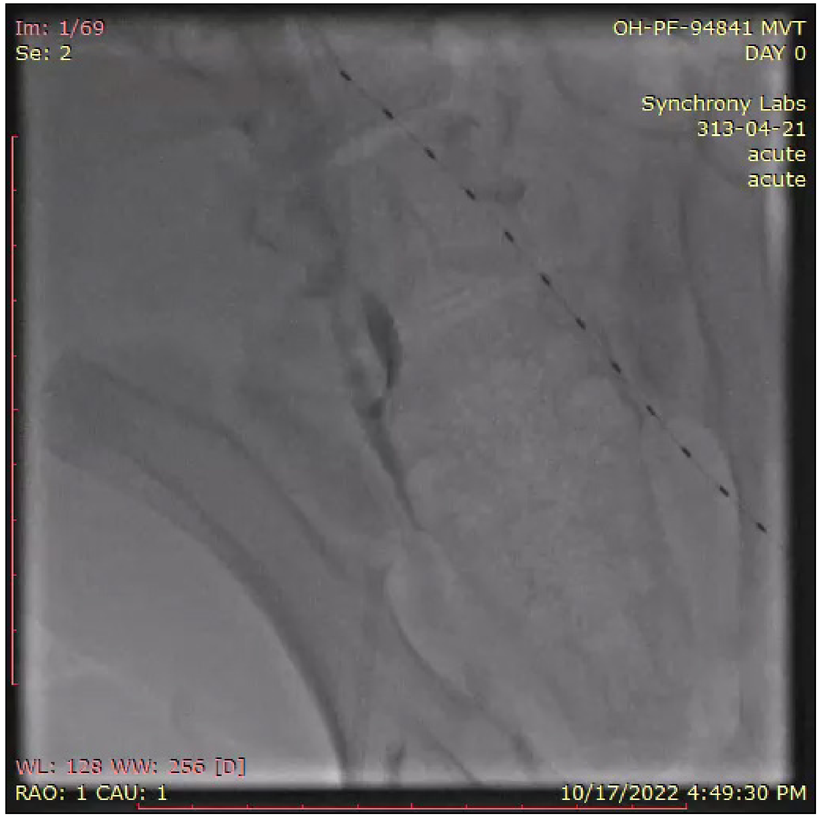

Pig #	Location	Treatment	Ultrasound
22	Right Iliac Vein	MVT-101 and tPA	Endovascular

Pre Treatment

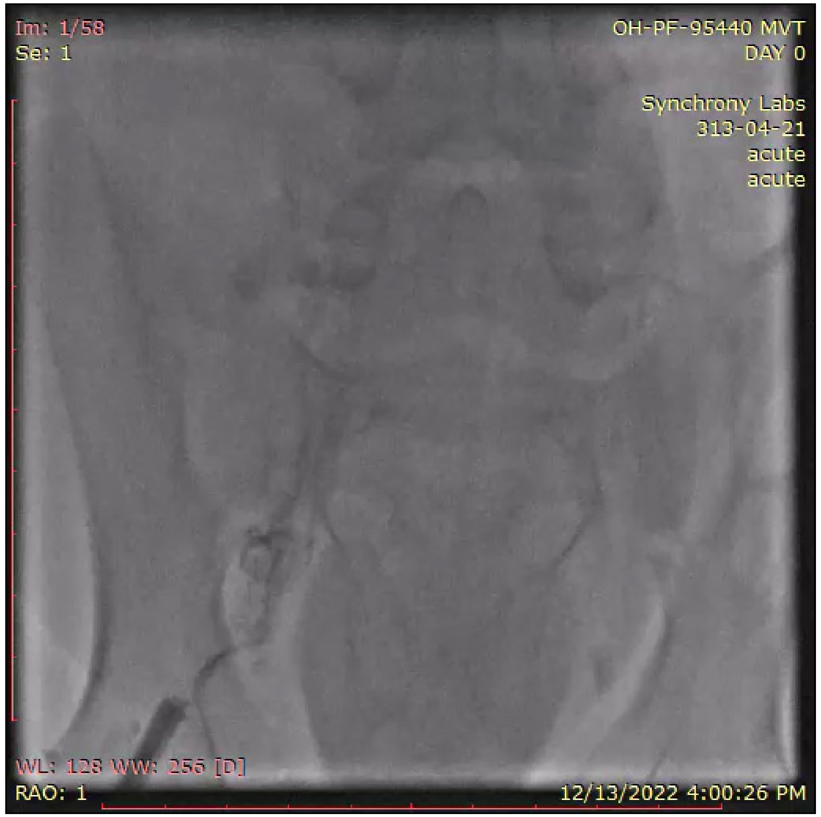

Post Treatment

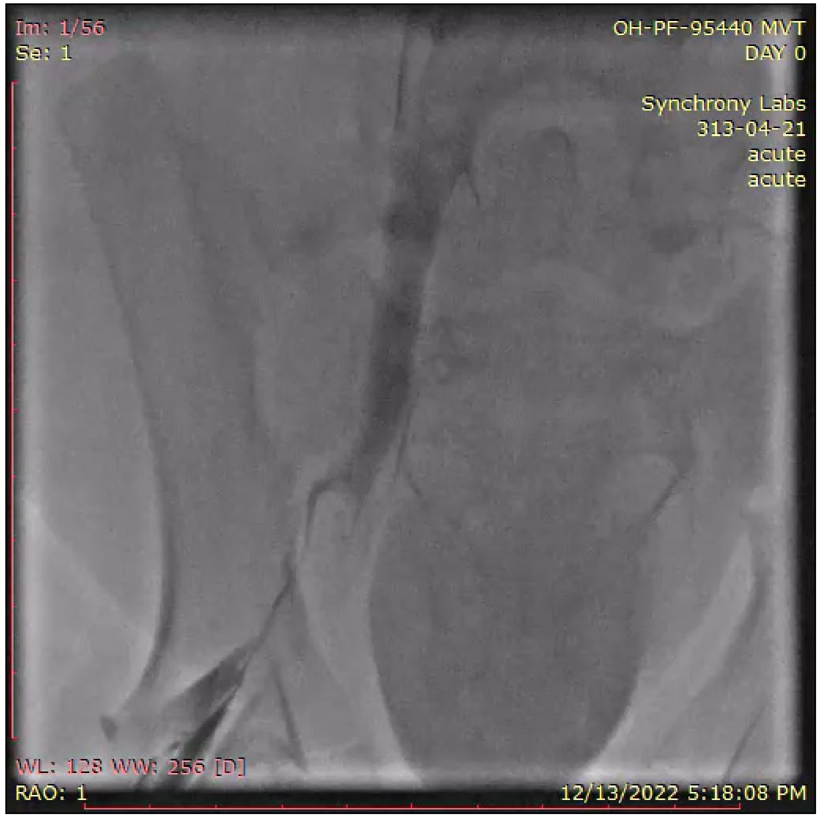

Pig #	Location	Treatment	Ultrasound
24	Left Iliac Vein	tPA	Endovascular

Pre Treatment

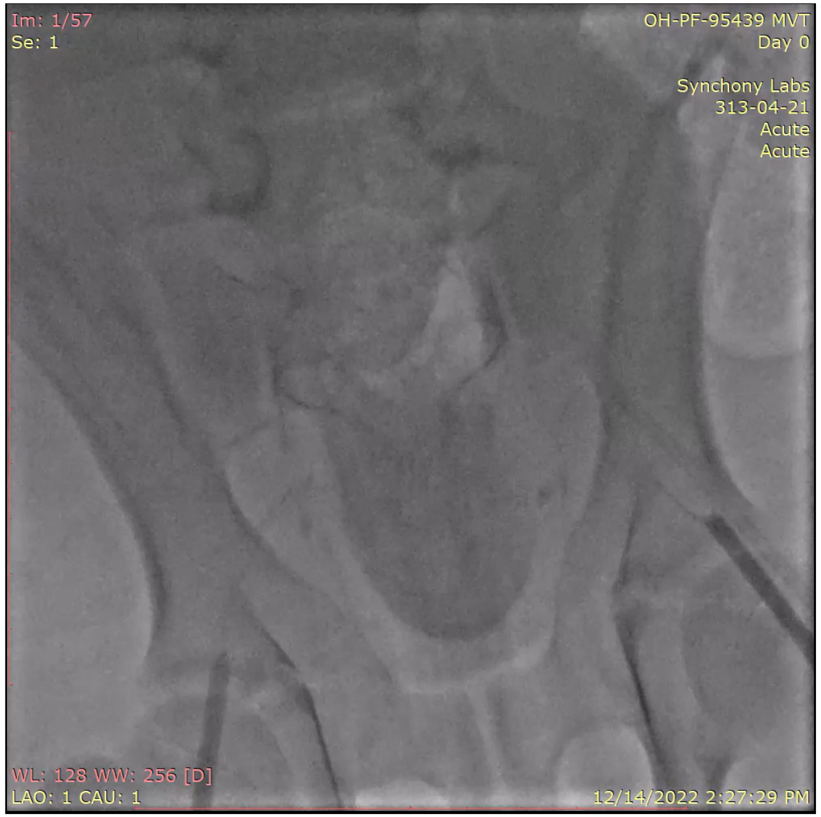

Post Treatment

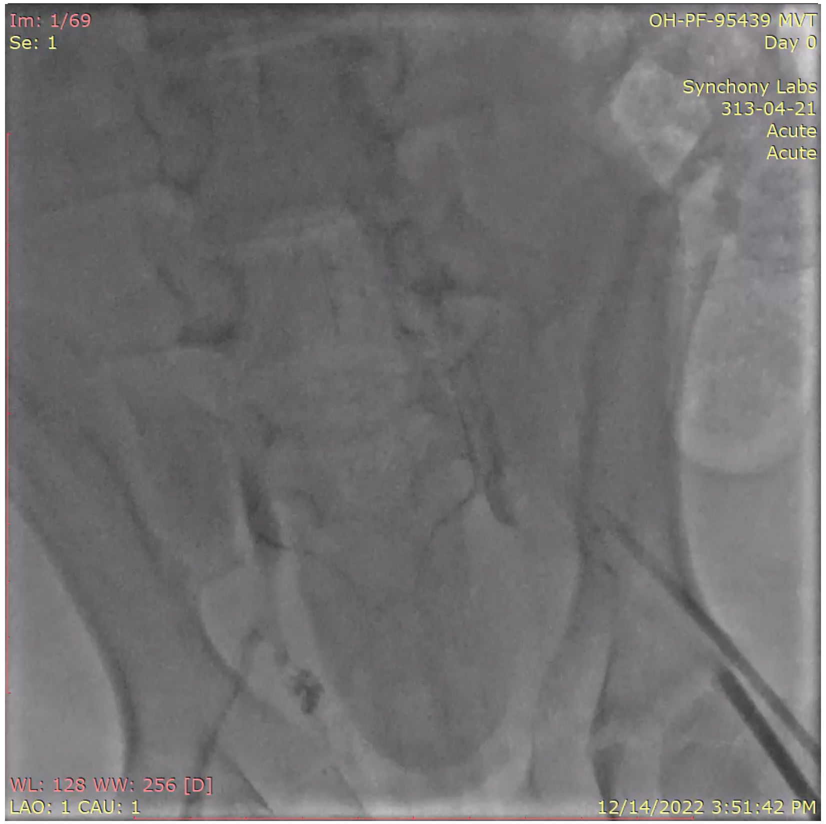

Pig #	Location	Treatment	Ultrasound
16	Left Iliac Vein	No Treatment	Endovascular

Pre Treatment

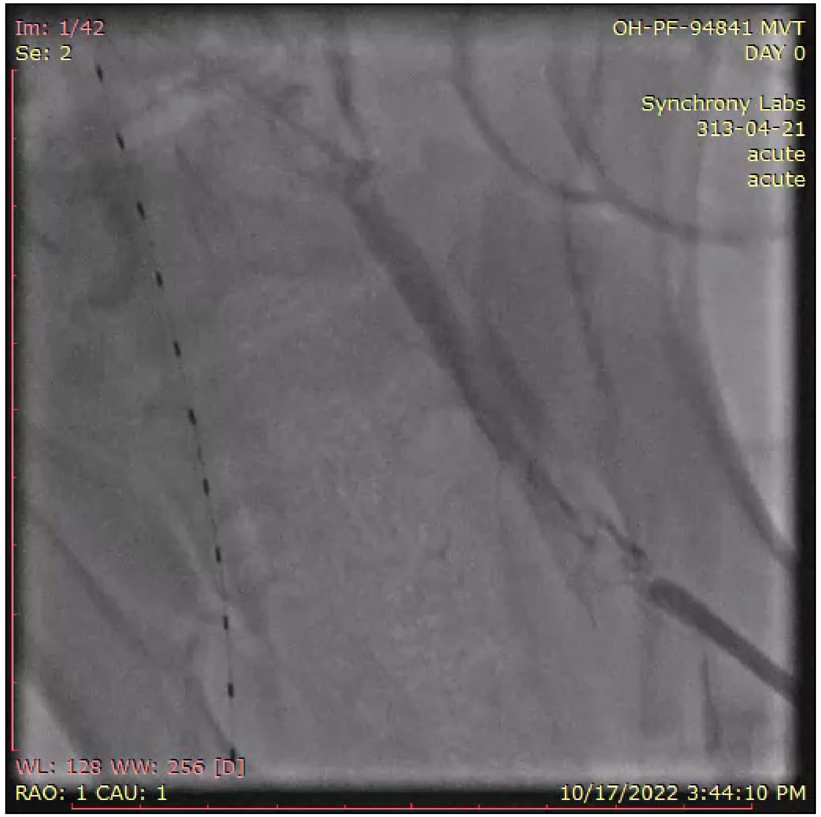

Post Treatment

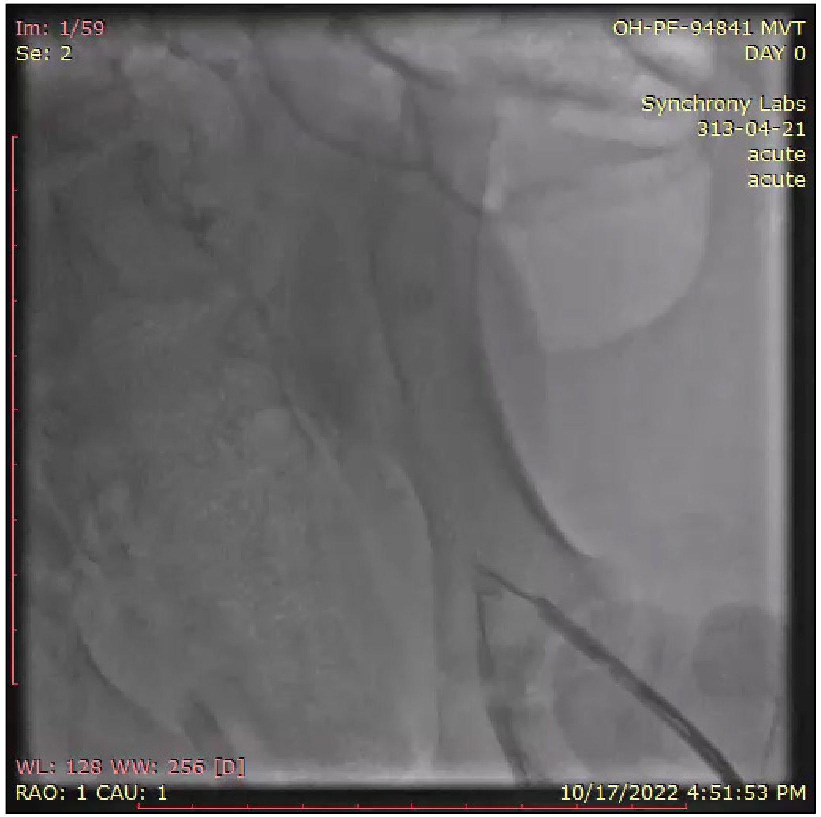

Transcutaneous

Pig #	Location	Treatment	Ultrasound
13	Left Iliac Vein	MVT-101 with tPA	Transcutaneous

Pre Treatment

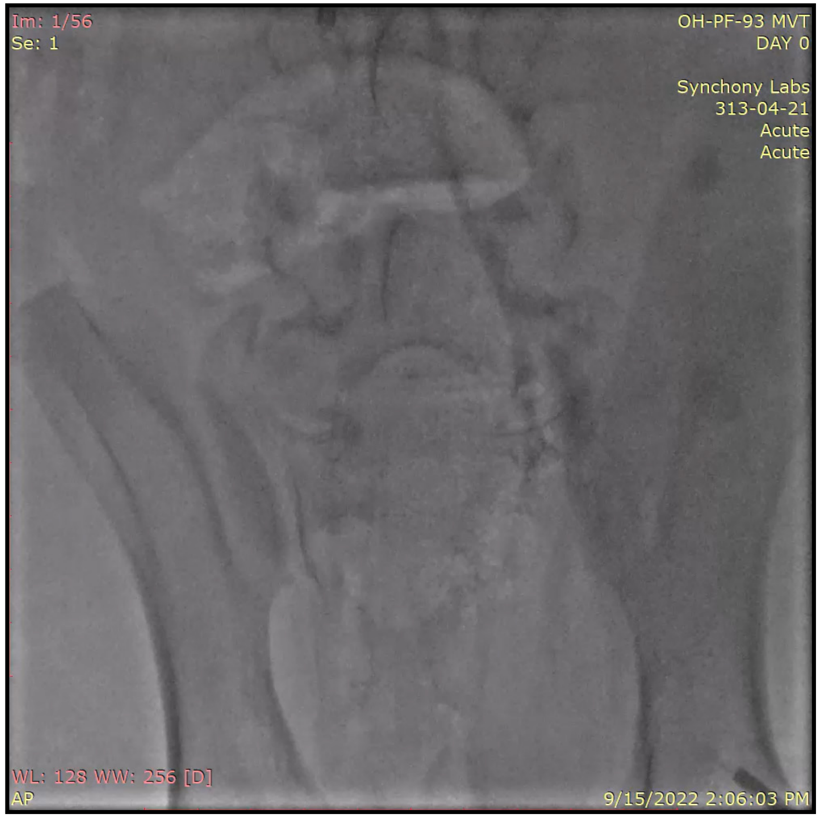

Post Treatment

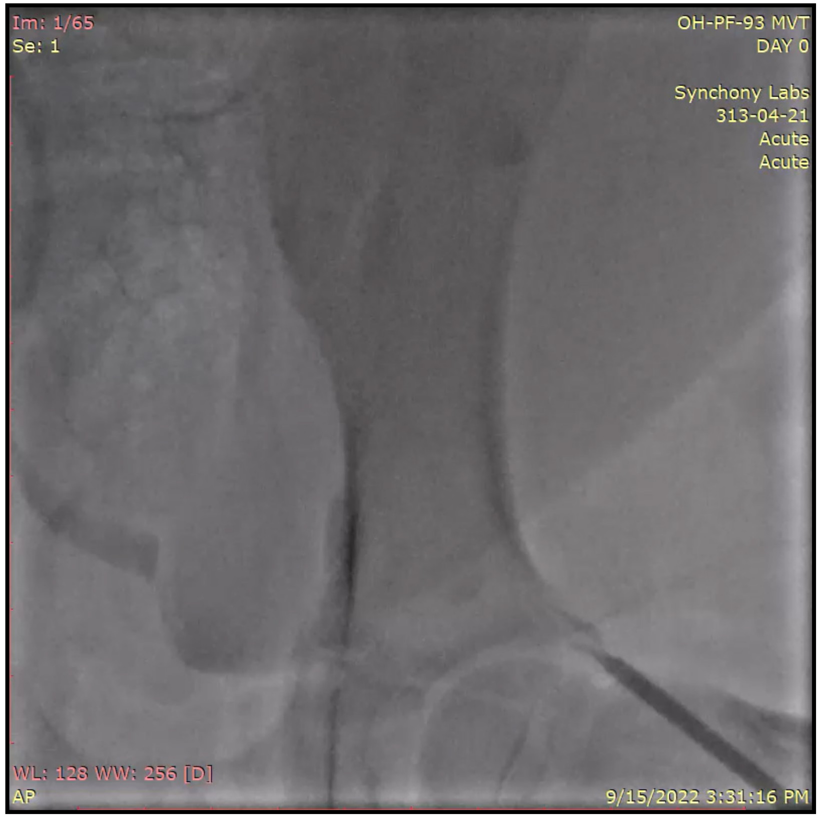

Pig #	Location	Treatment	Ultrasound
29	Right Iliac Vein	tPA	Transcutaneous

Pre Treatment

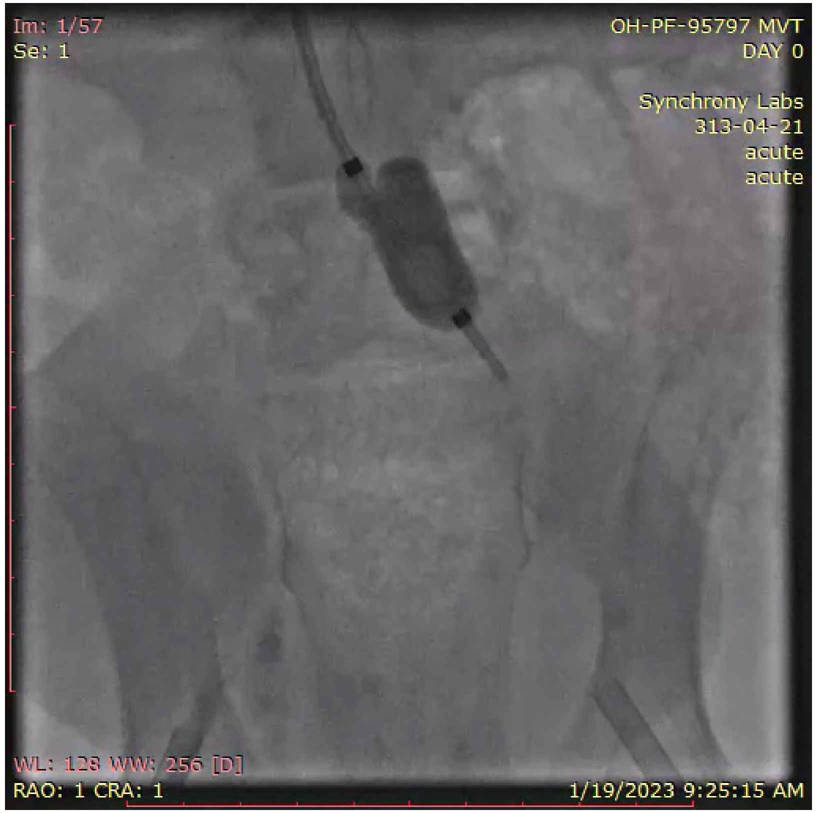

Post Treatment

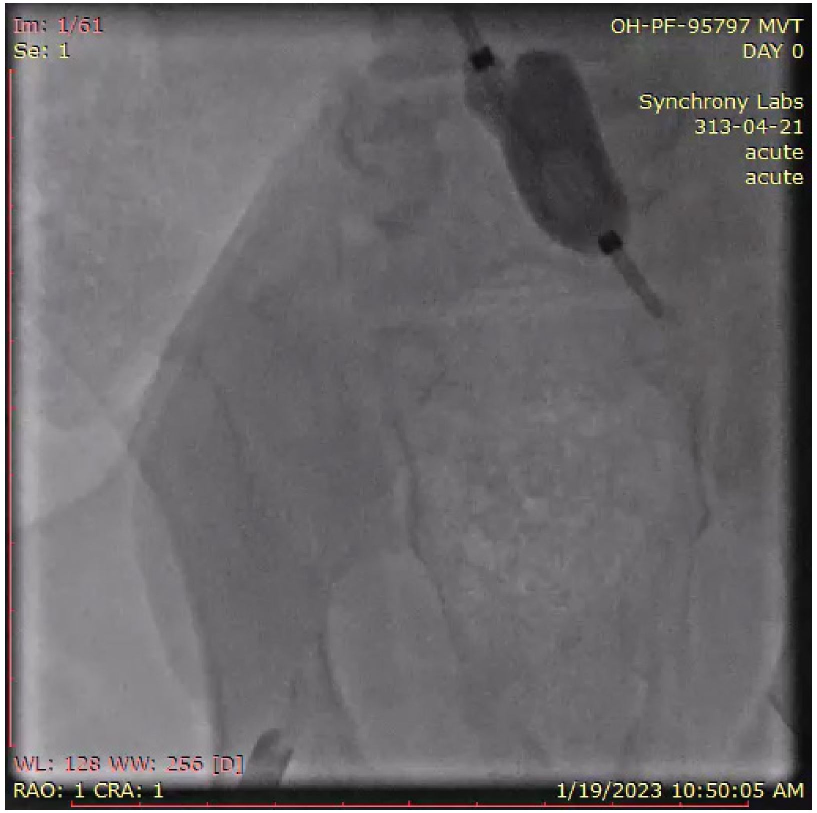

Pig #	Location	Treatment	Ultrasound
14	Right Iliac Vein	No Treatment	Transcutaneous

Pre Treatment

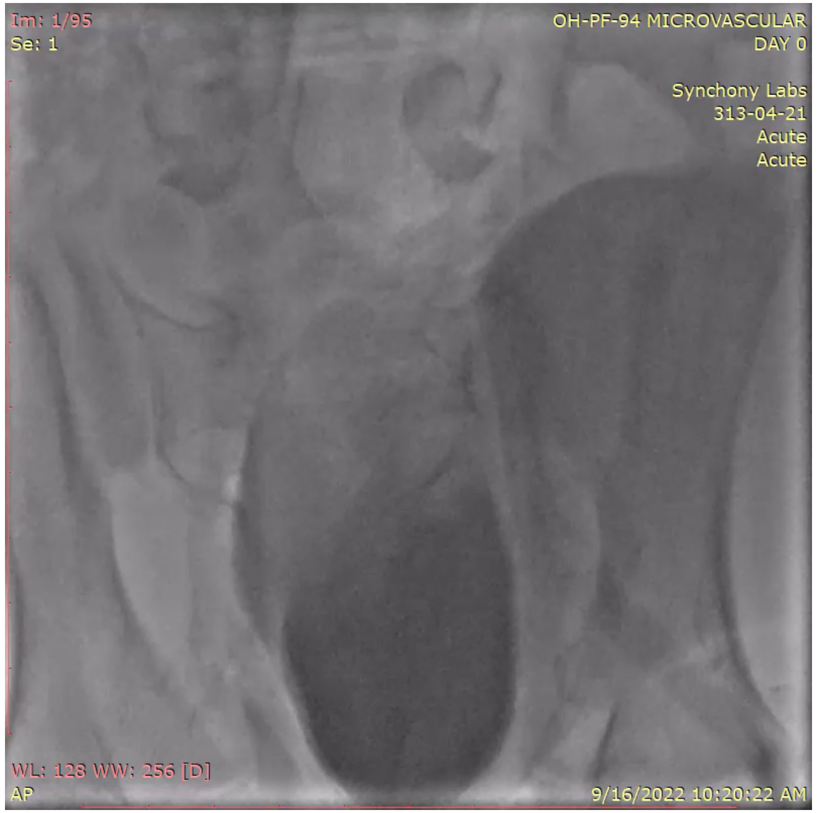

Post Treatment

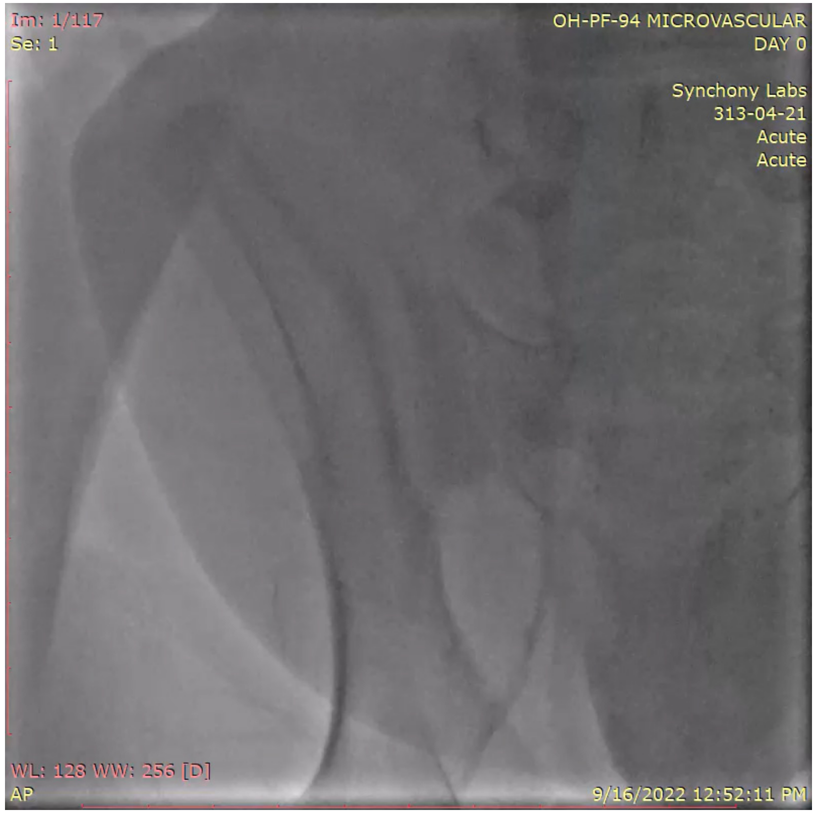

Supplement: Supplementary file 1 — Supplementary figures. [file ntnov09p0289s1.pdf]
